# Supplementary material for: Glutamate production from aerial nitrogen using the nitrogen-fixing bacterium Klebsiella oxytoca
Source: Commun Biol. 2024 Apr 11;7:443. doi: 10.1038/s42003-024-06147-z (PMC11009414; doi:10.1038/s42003-024-06147-z)
Supplement: Supplementary file 2 — Supplementary Information [file 42003_2024_6147_MOESM2_ESM.pdf]

Inventory of Supplementary Information

Glutamate production from aerial nitrogen using the nitrogen-fixing bacterium *Klebsiella oxytoca*

Daisuke Yoshidome, Makoto Hidaka, Toka Miyanaga, Yusuke Ito, Saori Kosono, and Makoto Nishiyama

Supplementary Fig. 1. Effect of nitrogen compounds on nitrogenase activity and cell growth of *K. oxytoca* NG13.

Supplementary Fig. 2. Effect of organic acids combined with glucose on nitrogenase activity and cell growth of *K. oxytoca* NG13 and CgCS strain, and glutamate production of CgCS strain.

Supplementary Fig. 3. Effect of organic acids alone on nitrogenase activity and cell growth of *K. oxytoca* NG13.

Supplementary Fig. 4. Effect of various concentrations of citrate on nitrogenase activity and cell growth of *K. oxytoca* NG13 and CgCS strain, and glutamate production of CgCS strain.

Supplementary Fig. 5. Properties of *K. oxytoca* NG13 in KDC medium.

Supplementary Fig. 6. Effect of heterologous expression of various citrate synthase and 2-metylcitrate synthase genes in *K. oxytoca* NG13.

Supplementary Fig. 7. Effect of culture volumes on nitrogenase activity, cell growth, and glutamate production of CgCS strain.

Supplementary Fig. 8. Extracellular glutamate production by CitS, CgCS and CgCS+CitS strains in ϕ18 test tube.

Supplementary Fig. 9. Extracellular glutamate production by CitS, CgCS and CgCS+CitS strains in non-diazotrophic condition supplemented with 1 g L^{-1} NH_4Cl.

Supplementary Fig. 10. Sequence of the open reading frame encoding a putative CS in *K. oxytoca* NG13 (KoCS).

Supplementary Fig. 11. Sequence of the open reading frame encoding a putative CitS in *K. oxytoca* NG13.

Supplementary Table 1. Plasmids used in this study.

Supplementary Table 2. Oligonucleotide primers used in this study.

Supplementary References

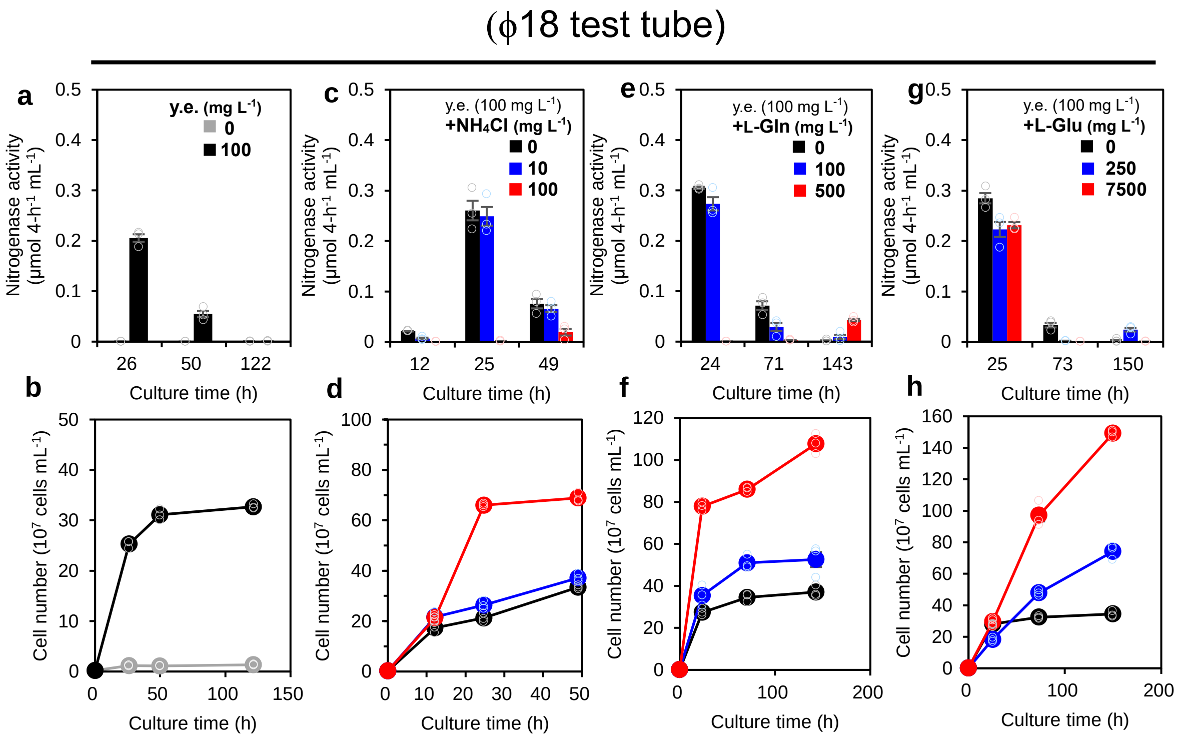

Supplementary Fig. 1. Effect of nitrogen compounds on nitrogenase activity and cell growth of *K. oxytoca* NG13.

K. oxytoca NG13 were cultured statically under air in non-hermetic ϕ18 test tubes containing 5 mL of KDC medium without yeast extract (y.e.) (**a, b**) and KDC medium supplied with NH_4Cl (**c, d**), L-glutamine (**e, f**), and L-glutamate (**g, h**) at indicated concentrations. Nitrogenase activity (**a, c, e, g**) and cell number (**b, d, f, h**) were measured at each time point using independent triple biological replicates. Data are shown as the mean of three biological replicates with standard deviation.

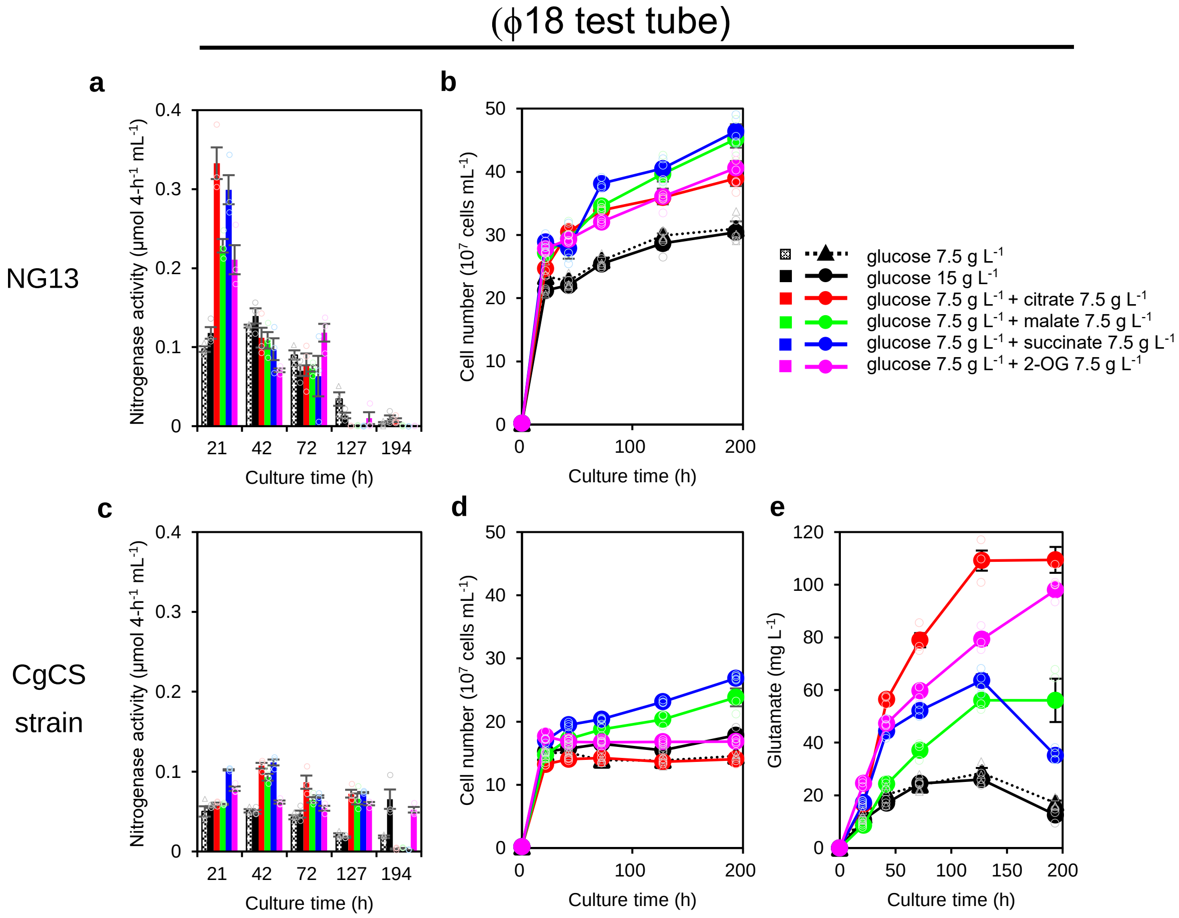

Supplementary Fig. 2. Effect of organic acids combined with glucose on nitrogenase activity and cell growth of *K. oxytoca* NG13 and CgCS strain, and glutamate production of CgCS strain.

K. oxytoca NG13 (**a, b**) and CgCS strain (**c, d, e**) were cultured statically under air in non-hermetic ϕ18 test tubes containing 5 mL of Rennie salt basal medium[1] supplemented with glucose plus citrate (red), glucose plus malate (green), glucose plus succinate (blue) at each concentration of 7.5 g L^{-1}, and only glucose of 7.5 g L^{-1} (dotted black) and 15 g L^{-1} (solid black). Nitrogenase activity (**a, c**), cell number (**b, d**), and extracellular glutamate production (**e**) were measured at each time point using independent triple biological replicates. Data are shown as the mean of three biological replicates with standard deviation.

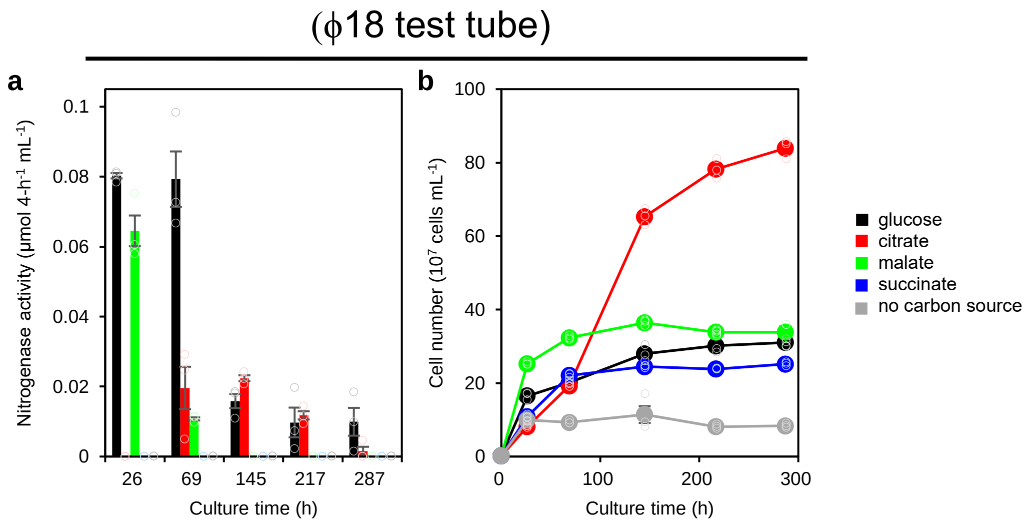

Supplementary Fig. 3. Effect of organic acids alone on nitrogenase activity and cell growth of *K. oxytoca* NG13.

K. oxytoca NG13 was cultured statically under air in non-hermetic ϕ18 test tubes containing 5 mL of Rennie salt basal medium[1] supplemented with glucose (black), citrate (red), malate (green), and succinate (blue) at 15 g L^{-1}, and without carbon source (grey). Nitrogenase activity (**a**) and cell number (**b**) were measured at each time point using independent triple biological replicates. Data are shown as the mean of three biological replicates with standard deviation. Nitrogenase activity was not detected at all measurement points in condition of succinate (blue) and no carbon source (grey).

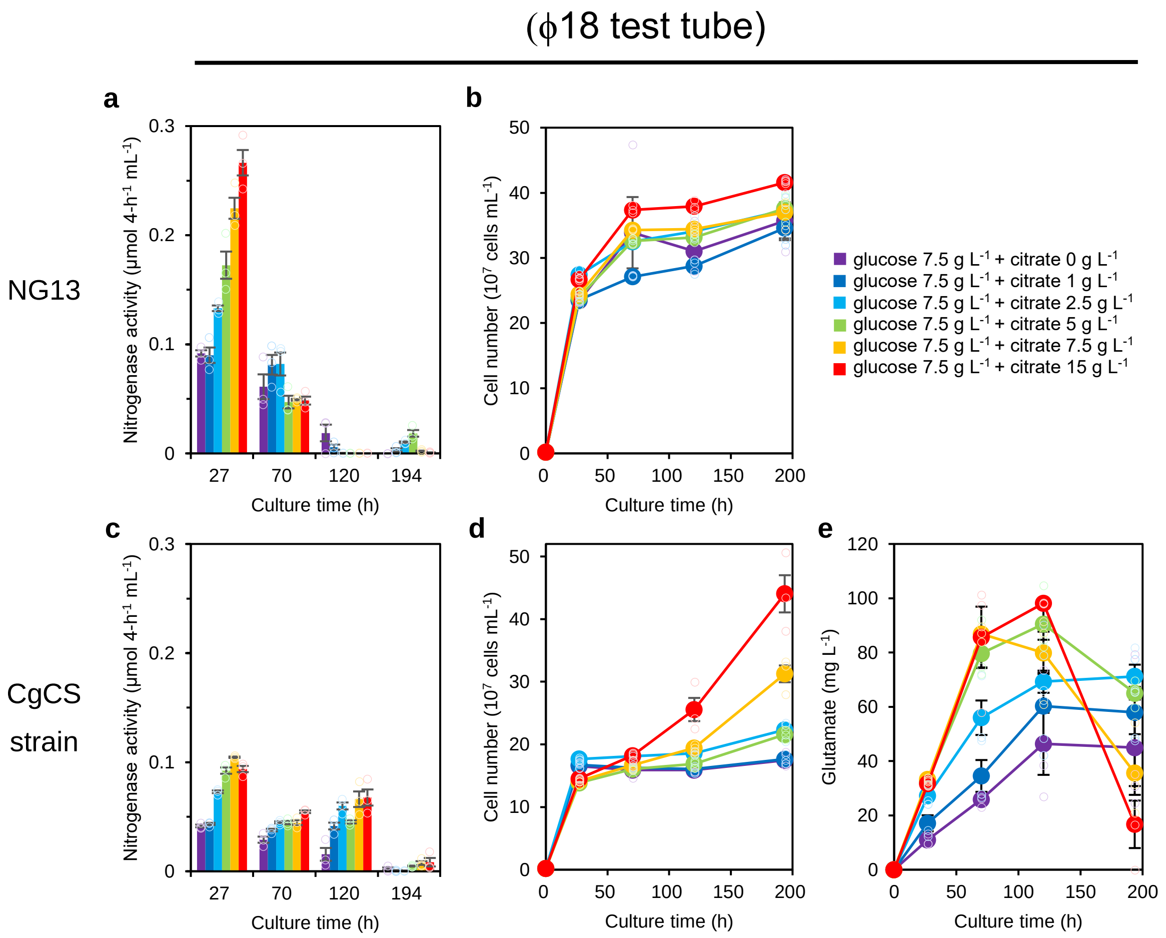

Supplementary Fig. 4. Effect of various concentrations of citrate on nitrogenase activity and cell growth of *K. oxytoca* NG13 and CgCS strain, and glutamate production of CgCS strain.

K. oxytoca NG13 (**a, b**) and CgCS strain (**c, d, e**) were cultured statically under air in non-hermetic ϕ18 test tubes containing 5 mL of Rennie salt basal medium[1] supplemented with 7.5 g L^{-1} of glucose and various concentrations of citrate. Nitrogenase activity (**a, c**), cell number (**b, d**), and extracellular glutamate production (**e**) were measured at each time point using independent triple biological replicates. Data are shown as the mean of three biological replicates with standard deviation.

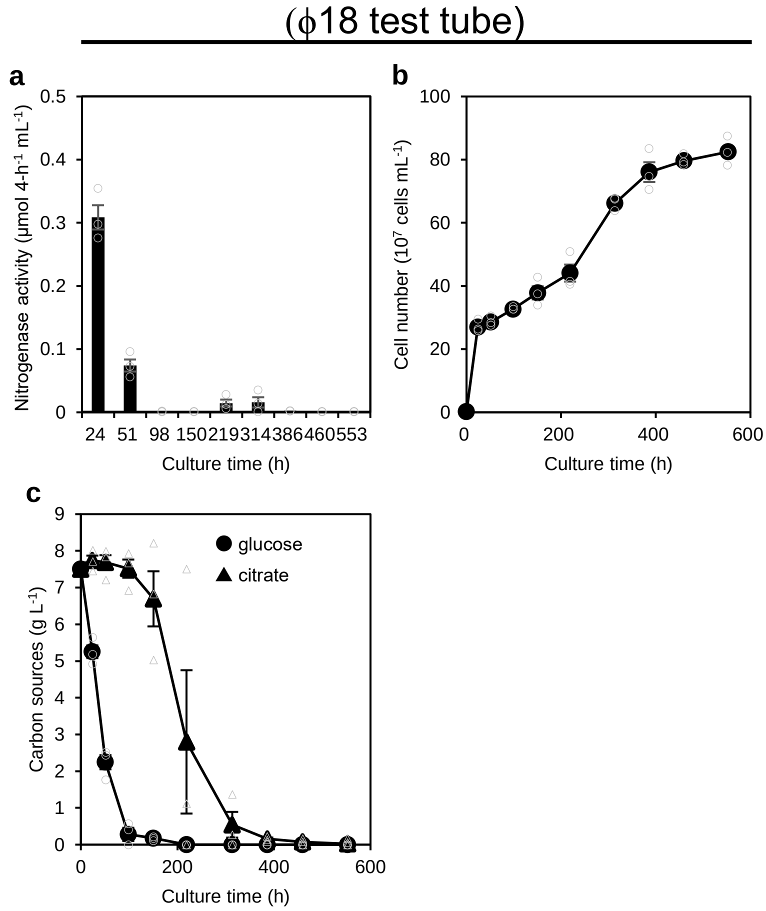

Supplementary Fig. 5. Properties of *K. oxytoca* NG13 in KDC medium.

K. oxytoca NG13 was cultured statically under air in non-hermetic ϕ18 test tubes containing 5 mL of KDC medium. Nitrogenase activity (**a**), cell number (**b**), and carbon source consumption (**c,** glucose: circle, citrate: triangle) were measured at each time point using independent triple biological replicates. Data are shown as the mean of three biological replicates with standard deviation.

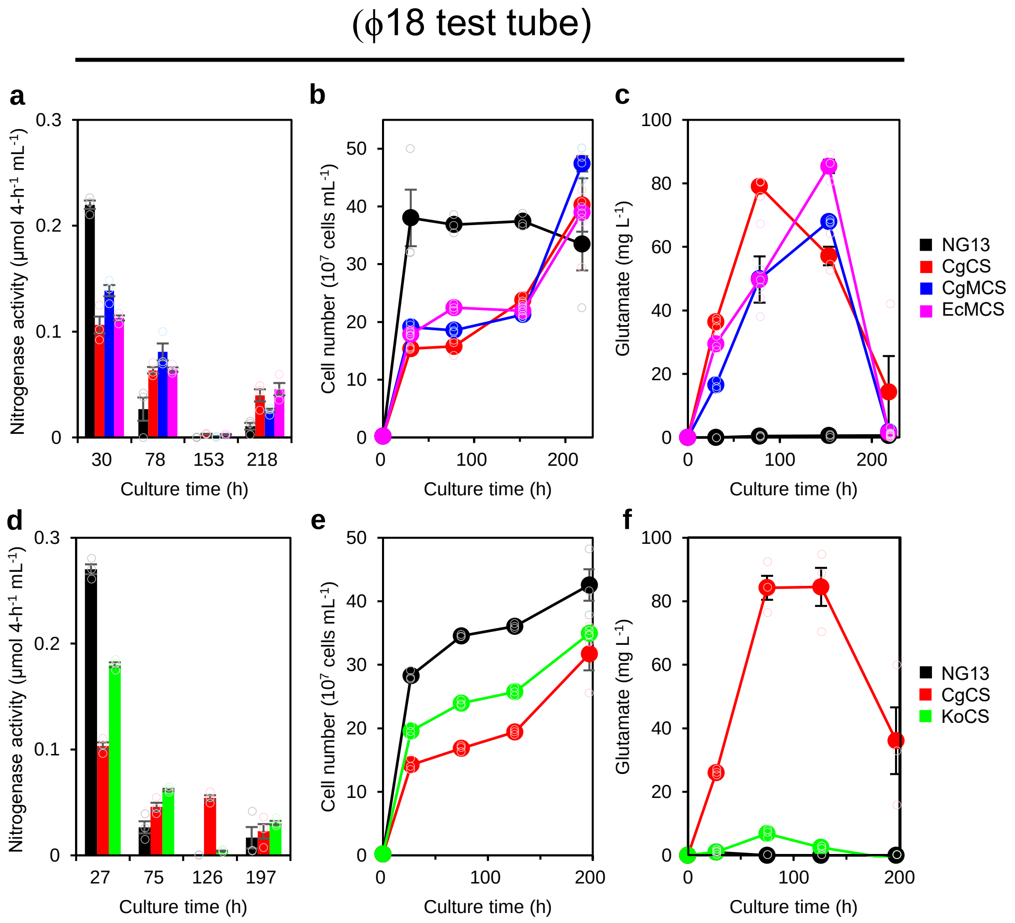

Supplementary Fig. 6. Effect of heterologous expression of various citrate synthase and 2-metylcitrate synthase genes in *K. oxytoca* NG13.

K. oxytoca NG13 (black), CgCS strain (red), and the strains overproducing 2-metylcitrate synthase from *Corynebacterium glutamicum* (CgMCS strain, blue), *Escherichia coli* (EcMCS strain, pink), or citrate synthase from NG13 (KoMCS strain, green) were cultured statically under air in non-hermetic ϕ18 test tubes containing 5 mL of KDC medium. Nitrogenase activity (**a, d**), cell number (**b, e**), and extracellular glutamate production (**c, f**) were measured at each time point using independent triple biological replicates. Data are shown as the mean of three biological replicates with standard deviation.

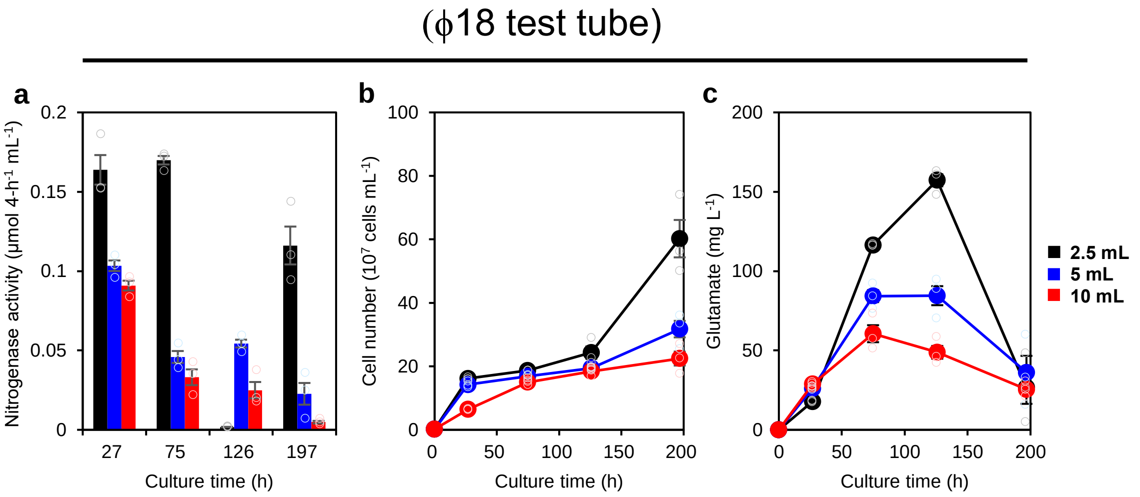

Supplementary Fig. 7. Effect of culture volumes on nitrogenase activity, cell growth, and glutamate production of CgCS strain.

CgCS strain was cultured statically under air in non-hermetic ϕ18 test tubes containing KDC medium of 2.5 mL (black), 5 mL (blue), and 10 mL (red). Nitrogenase activity (**a**), cell number (**b**), and extracellular glutamate production (**c**) were measured at each time point using independent triple biological replicates. Data are shown as the mean of three biological replicates with standard deviation.

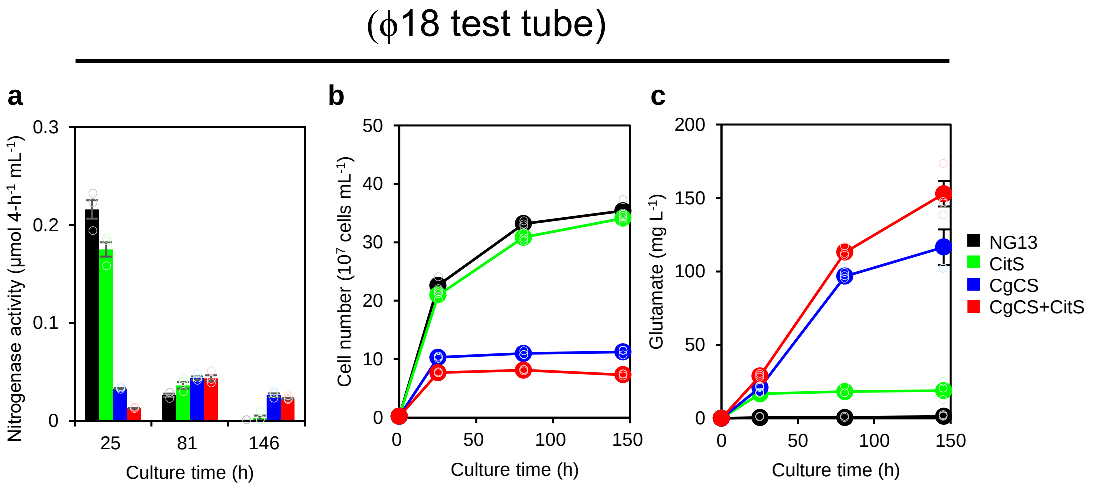

Supplementary Fig. 8. Extracellular glutamate production by CitS, CgCS and CgCS+CitS strains in ϕ18 test tube.

K. oxytoca NG13 (black), CitS (green), CgCS (blue), and CgCS+CitS (red) strains were cultured statically under air in non-hermetic ϕ18 test tubes containing 5 mL of KDC medium. Nitrogenase activity (**a**), cell number (**b**), and extracellular glutamate production (**c**) were measured at each time point using independent triple biological replicates. Data are shown as the mean of three biological replicates with standard deviation.

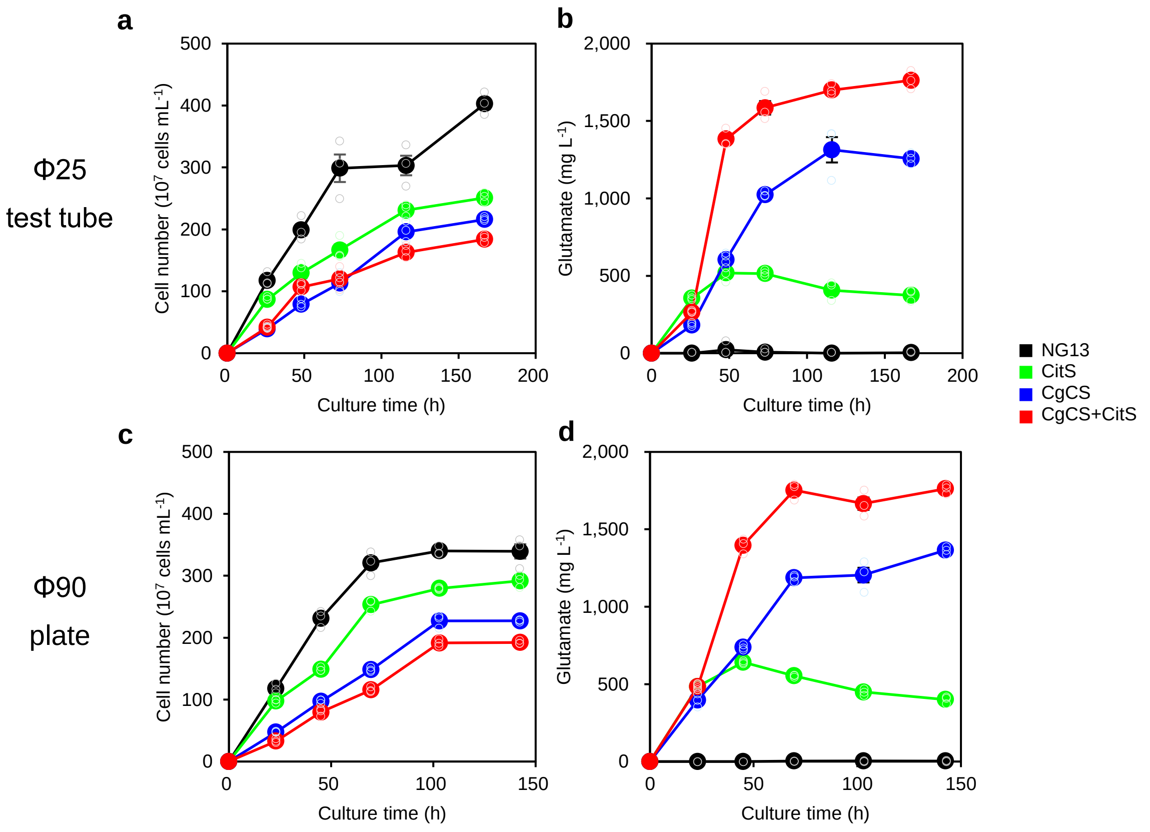

Supplementary Fig. 9. Extracellular glutamate production by CitS, CgCS and CgCS+CitS strains in non-diazotrophic condition supplemented with 1 g L^{-1} NH_4Cl. *K. oxytoca* NG13 (black), CitS (green), CgCS (blue), and CgCS+CitS (red) strains were cultured in KDC medium supplemented with 1 g L^{-1} NH_4Cl. Cultures were performed statically under air in 5 mL using non-hermetic ϕ25 test tubes (**a**, **b**) and 25 mL using non-hermetic ϕ90 plates (**c**, **d**). Cell number (**a**, **c**) and extracellular glutamate production (**b**, **d**) were measured at each time point using independent triple biological replicates. Data are shown as the mean of three biological replicates with standard deviation.

Supplementary Fig. 10. Nucleotide sequence of the open reading frame encoding a putative CS in *K. oxytoca* NG13 (KoCS).

ATGTCTGATGCTAAAGCAAAAATCACCCTGGGTGGTGATACTGCTATCGAACTGGATGTGCTAAAGG
GCACGCTCGGTCAGGATGTTATTGATATTCGTAGTCTTGGTTCAAAAGGCGTATTTACTTTTGACCC
TGGTTTCACCTCAACCGCATCCTGCGAATCTAAAATCACCTTTATCGACGGTGATGAAGGTATCCTG
CTGCACCGCGGTTTTCCGATCGATCAGTTAGCAACCGACTCCAACTATCTGGAAGTATGCTACATCC
TGCTGAACGGTGAGAAGCCGACTCAGGCCCAGTACGACGAATTCAAAACAATCGTTACTCGCCACAC
TATGATTCACGAACAGATTACCCGTCTGTTCCACGCGTTCCGCCGCGATTCACATCCGATGGCCGTT
ATGTGCGGTATCACCGGCGCGCTGGCCGCGTTCTATCACGATTCGCTGGATGTGAATAACCCACGCC
ATCGCGATATCGCCGCATTCCGCCTGCTCTCCAAGATGCCGACGATGGCGGCAATGTGTTACAAATA
TTCTATCGGTCAGCCTTTCGTTTATCCGCGCAACGACCTCTCCTACGCGGGTAACTTCCTGAATATG
ATGTTCTCCACGCCGTGTGAAAAATATGAAGTGAACCCGATTCTGGAACGCGCGATGGACCGTATCC
TGATCCTGCACGCCGACCACGAACAAAACGCTTCGACCTCCACCGTGCGTACCGCCGGCTCTTCCGG
CGCGAACCCGTTTGCCTGCATCGCAGCGGGCATTGCCTCCCTGTGGGGACCGGCGCACGGCGGCGCC
AACGAAGCGGCGCTGAAGATGCTCGAAGAGATCAGCTCCGTTGAACACATTCCGGAATTCGTTCGTC
GCGCGAAAGACAAAAACGACTCTTTCCGCCTGATGGGCTTTGGCCACCGGGTGTACAAAAACTACGA
CCCGCGCGCCACCGTTATGCGTGAAACCTGCCATGAAGTGCTGAAAGAGCTGGGCACCAAAGACGAT
CTGCTGGAAGTGGCTATGGAGCTTGAGCACATCGCGCTTAACGACCCGTACTTCATTGAGAAGAAAC
TTTACCCTAACGTCGATTTCTACTCCGGTATCATCCTCAAAGCGATGGGCATCCCATCCTCCATGTT
TACCGTTATCTTCGCGATGGCGCGTACCGTGGGCTGGATTGCGCACTGGAACGAAATGCACAGCGAC
GGCATGAAAATAGCCCGTCCGCGTCAGCTGTATACCGGCTACGAAAAGCGCGATTTCAAAAACGATA
TCGCCCGCTAA

Supplementary Fig. 11. Nucleotide sequence of the open reading frame encoding a putative CitS in *K. oxytoca* NG13.

ATGACTAATATGAGCCAGGCTCCATCTGCAGAGAAAAAAGGCGTTAGCGATATTCTGGGGTTTAAAA
TCTTCGGCATGCCGCTACCGCTTTACGCCTTTGCGTTAATCACTTTACTACTTTCACACTTTTATAA
TGCCCTGCCGACCGACATTGTCGGCGGCTTCGCCATCATGTTTATTATTGGCGCCGTTTTTGGAGAA
ATTGGCAAACGCCTGCCGATCTTCAATAAATATATCGGCGGCGCGCCGGTGATGATCTTCCTCGTGG
CCGCCTATTTCGTTTATGCAGGCATTTTCACCCAGAAAGAAATTGATGCGATCACTAATGTGATGGA
TAAAAGTAACTTCCTGAACTTATTCATCGCAGTATTGATTACTGGTGCAATCCTCTCGGTTAACCGT
AAGCTGCTGCTGAAATCTCTGCTGGGTTATATTCCGACCATTTTAATGGGGATCCTCGGCGCATCCA
TCTTCGGGATTCTCATCGGCTTGTGCTTCGGTATCTCCATCGACCGCATTATGATGCTGTACGTCCT
GCCGATTATGGGTGGCGGCAACGGCGCGGGCGCGGTGCCGCTATCTGAAATTTATCACTCCGTCACC
GGCCGTTCGCGTGAAGAGTACTACTCCACCGCGATTGCTATCCTGACCATCGCCAACATCTTTGCCA
TCGTGTTTGCCGCGCTGCTCGATATTATCGGTAAAAAGCACACCTGGCTGAGCGGCGAAGGCGAGCT
GGTGCGCAAGGCCTCTTTCAAAGTAGAAGATGATGAAAAAGCGGGCCAGATTACCCATCGCGAAACG
GCCGTCGGCCTGGTGCTCGCCACAACCTGCTTCCTGCTGGCCTACGTTATCGCCAAGAAAATTCTGC
CAAGCATTGGCGGCGTATCTATCCACTATTTCGCCTGGATGGTGCTGATCGTCGCGGCGCTGAACGC
TTCCGGCCTCTGCTCGCCTGAGATTAAAGCTGGCGCTAAACGCCTATCTGACTTCTTCTCTAAGCAA
CTGCTGTGGGTACTGATGGTCGGCGTCGGCGTGTGCTACACCGACCTGCAGGAAATTATCGACGCGA
TTACCTTTGCGAACGTCATCATCGCGGCGGTGATCGTCGTGGGCGCGGTCATCGGCGCGGCCATCGG
CGGCTGGATGATTGGCTTCTTCCCGATTGAATCCGCGATCACCGCCGGTCTGTGCATGGCTAACCGC
GGCGGCTCGGGCGACCTGGAAGTACTCTCTGCCTGTAACCGTATGAATCTTATCTCTTATGCGCAAA
TCTCCTCCCGTCTGGGCGGCGGTATTGTGCTGGTCATTGCCAGCATCGTGTTCGGCATGATGATGTA
A

Supplementary Table 1. Plasmids used in this study.

Name	Description
pCCS	Expression of CgCS gene under the *trc* promoter, pSC101 *ori*, Tet^{R}
pCMCS	Expression of CgMCS gene under the *trc* promoter, pSC101 *ori*, Tet^{R}
pEMCS	Expression of EcMCS gene under the *trc* promoter, pSC101 *ori*, Tet^{R}
pKCS	Expression of KoCS gene under the *trc* promoter, pSC101 *ori*, Tet^{R}
pKCT	Expression of CitS gene under the *trc* promoter, pCDF *ori*, Spc^{R}

Supplementary Table 2. Oligonucleotide primers used in this study.

	Sequence (5’ to 3’)	Amplification of
1	CGATTAAATAAGGAGGAATAAACCATGTTTGAAAGGGATATC	CgCS gene
2	CGGGTACCGAGCTCGAATTCTTAGCGCTCCTCGCGAGG	CgCS gene
3	GAATTCGAGCTCGGTACC	pMW118
4	GGTTATTGTCTCATGAGCGG	pMW118
5	CCGCTCATGAGACAATAACCAATTCTCATGTTTGACAG	Tet^R cassette of 707-FLPe
6	GGATCTTCACCTAGATCCTTATTCAGGTCGAGGTGGCCCG	Tet^R cassette of 707-FLPe
7	AAGGATCTAGGTGAAGATCC	pMW118
8	GGGTGTTGGCGGGTGTCGGGCGCTCACTGCCCGCTTTC	pMW118
9	CCCGACACCCGCCAACACCC	*lacI* and P_{trc} region of pTrcHis2-TOPO/lacZ
10	GGTTTATTCCTCCTTATTTAATCG	*lacI* and P_{trc} region of pTrcHis2-TOPO/lacZ
11	CGATTAAATAAGGAGGAATAAACCATGTCCAGCGCCACAACC	CgMCS gene
12	CGGGTACCGAGCTCGAATTCTTAACGCTTTTCAATGGG	CgMCS gene
13	CGATTAAATAAGGAGGAATAAACCATGAGCGACACAACGATC	EcMCS gene
14	CGGGTACCGAGCTCGAATTCTTACTGGCGCTTATCCAG	EcMCS gene
15	CGATTAAATAAGGAGGAATAAACCATGTCTGATGCTAAAGCA	KoCS gene

16	CGGGTACCGAGCTCGAATTCTTAGCGGGCGATATCGTT	KoCS gene
17	CGATTAAATAAGGAGGAATAAACCATGACTAATATGAGC CAGGC	CitS gene
18	CGCTAGTAGACGAGTCCATGTTACATCATCATGCCGAAC	CitS gene
19	CATGGACTCGTCTACTAG	*lacI*-pCDF *ori*-Spc^R region of pCDFDuet
20	AACATTATCCAGAACGGGAGTCCTAATGCAGGAGTCGC	*lacI*-pCDF *ori*-Spc^R region of pCDFDuet
21	CTCCCGTTCTGGATAATG	P_{trc} region of pTrcHis2-TOPO/lacZ

Supplementary References

1. Rennie, R. J. A single medium for the isolation of acetylene-reducing (dinitrogen-fixing) bacteria from soils. *Can. J. Microbiol.* **27**, 8–14 (1981).
