# Supplementary material for: Glutamate production from aerial nitrogen using the nitrogen-fixing bacterium Klebsiella oxytoca
Source: Commun Biol. 2024 Apr 11;7:443. doi: 10.1038/s42003-024-06147-z (PMC11009414; doi:10.1038/s42003-024-06147-z)
Supplement: Supplementary file 3 — Description of Additional Supplementary Materials [file 42003_2024_6147_MOESM3_ESM.docx]

**Description of Additional Supplementary Files**

**File name:** Supplementary Data

**Description:** All source data
